# Application of artificial scaffold systems in microbial metabolic engineering

**DOI:** 10.3389/fbioe.2023.1328141

**Published:** 2023-12-22

**Authors:** Nana Liu, Wei Dong, Huanming Yang, Jing-Hua Li, Tsan-Yu Chiu

**Affiliations:** ^1^ College of Pharmaceutical Science, Zhejiang University of Technology, Hangzhou, China; ^2^ HIM-BGI Omics Center, Zhejiang Cancer Hospital, Hangzhou Institute of Medicine (HIM), Chinese Academy of Sciences (CAS), Hangzhou, China

**Keywords:** multienzyme complexes, enzyme molecular scaffolds, reaction microcompartments, microbial cell factory, synthetic scaffold

## Abstract

In nature, metabolic pathways are often organized into complex structures such as multienzyme complexes, enzyme molecular scaffolds, or reaction microcompartments. These structures help facilitate multi-step metabolic reactions. However, engineered metabolic pathways in microbial cell factories do not possess inherent metabolic regulatory mechanisms, which can result in metabolic imbalance. Taking inspiration from nature, scientists have successfully developed synthetic scaffolds to enhance the performance of engineered metabolic pathways in microbial cell factories. By recruiting enzymes, synthetic scaffolds facilitate the formation of multi-enzyme complexes, leading to the modulation of enzyme spatial distribution, increased enzyme activity, and a reduction in the loss of intermediate products and the toxicity associated with harmful intermediates within cells. In recent years, scaffolds based on proteins, nucleic acids, and various organelles have been developed and employed to facilitate multiple metabolic pathways. Despite varying degrees of success, synthetic scaffolds still encounter numerous challenges. The objective of this review is to provide a comprehensive introduction to these synthetic scaffolds and discuss their latest research advancements and challenges.

## 1 Introduction

Recently, the successful synthesis of diverse natural products has been achieved through the introduction of heterologous metabolic pathways into microbial cell factories (Srinivasan and Smolke, 2020; Yuan et al., 2022; Zhang et al., 2022). These synthetic pathways are collectively built with heterologous enzymes selected from various sources and are not accompanied by their regulatory partners in the new host (Tran et al., 2023). Thus, these unregulated enzymes may not be able to channel intermediates from the input reactions to the formation of end products properly (Dueber et al., 2009; Liu et al., 2023). In contrast, metabolic enzymes of a native pathway can be formed multi-enzyme complexes (Tittes et al., 2022), enzyme molecular scaffolds (Artzi et al., 2017), reaction microchambers [e.g., arom multienzyme complexes (Lumsden and Coggins, 1977), or caveolae, etc. (Polka et al., 2016)] to mediate the catalytic cascades coordinately. The structural entities play a pivotal role in facilitating efficient substrate transfer between adjacent enzyme active sites (Castellana et al., 2014). Thus, adopting a synthetic scaffold is one of the strategies to co-ordinate the none-native enzymes in microbial cell factories. To address the challenges several synthetic scaffolds have been devised for the precise modulation of enzyme activity. For instance, enzymes can be assembled on scaffolds made of DNA or protein, where protein-protein or DNA interactions are employed to facilitate the formation of cascading complexes among enzymes (Gad and Ayakar, 2021). These DNA or protein scaffolds are thought to form channels conducive to continuous metabolism, directing metabolic intermediates from one enzyme to another to regulate the spatial distribution of enzymes and increase their local concentrations (Tippmann et al., 2017). The utilization of synthetic scaffolds serves to significantly decrease interenzyme distances, thereby effectively restricting the diffusion of intermediate metabolitesand concurrently attenuates cellular cytotoxicity (Conrado et al., 2008). Though, the synthetic scaffolds had been successfully applied to metabolic engineering, trial and error are still the only way we may learn.

This review provides a detailed account of the applications of artificially synthesized scaffolds through specific case studies and comprehensively summarizes the latest advancements in various scaffold assembly methods. Additionally, it explores the potential challenges faced by artificially synthesized scaffolds. At the same time, the possible role of the current hot artificial intelligence (AI) technology in the application of artificial stent systems is also discussed.

## 2 Protein scaffold

Paired protein scaffolders fall into three main categories, including protein-peptide, peptide-peptide, and protein-protein pairs. These scaffolds can be fused directly with target enzymes to induce assembly, and they achieve enzyme assembly through non-covalent or covalent interactions between ligands and receptors in the scaffolds, with little effect on enzyme properties (Price et al., 2016; Chen et al., 2023).

### 2.1 Protein–peptide pair

**FIGURE 1:**
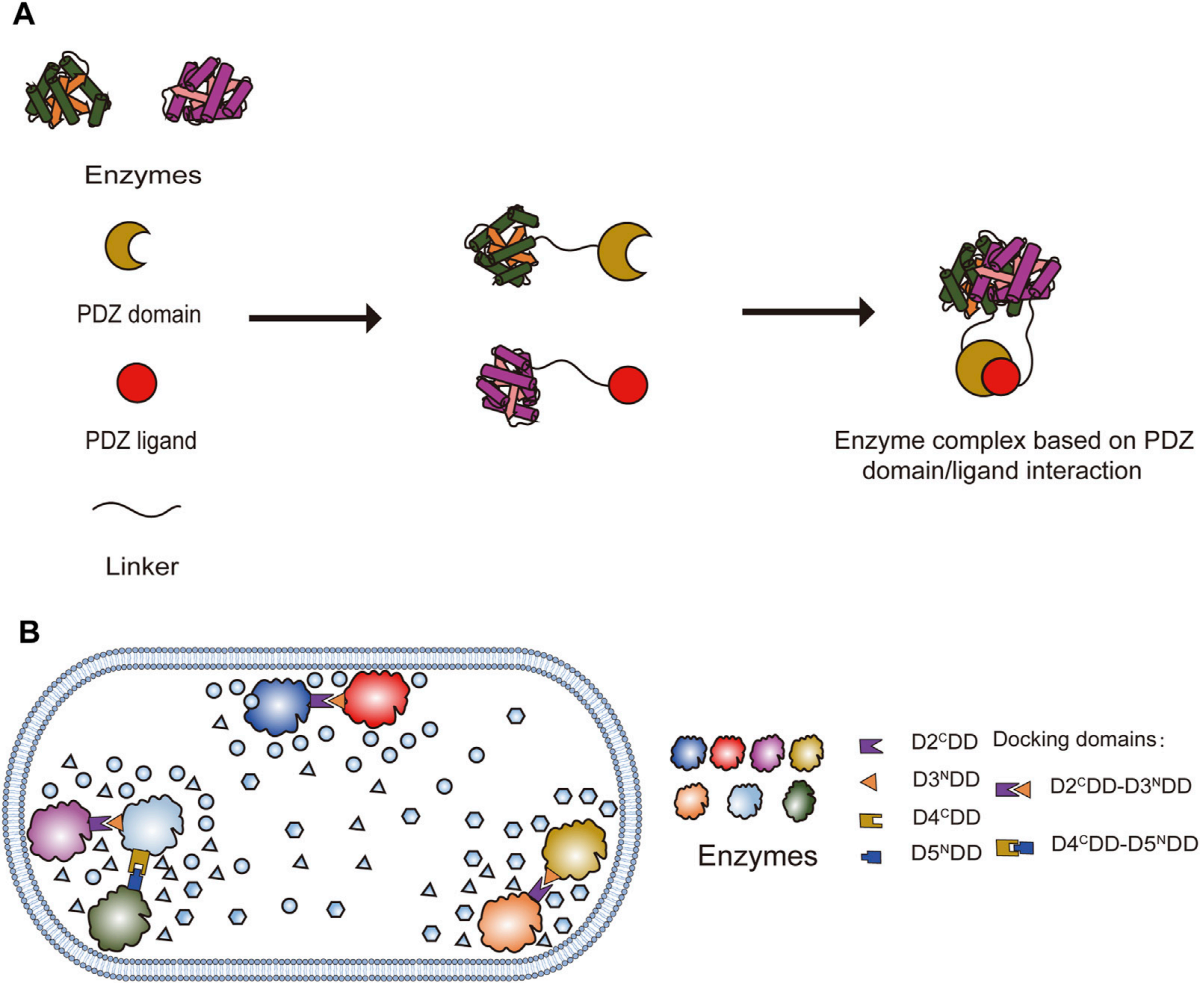
Protein scaffold; **(A)** Enzyme complex assembly based on PDZ domain and ligand interaction, adapted from (Gao et al., 2014); **(B)** Enzyme complex assembly based on mPKSeal strategy, adapted from (Sun et al., 2022).

Protein-peptide interaction recognition domains are widely present in various cells, where they participate in the assembly of intracellular complexes and play diverse cellular functions. Currently, several modular protein domains [e.g., PDZ domain, SH3 domains, GTPase binding domain (GBD), GBD_1_SH3_1_PDZ_2_, GBD_1_SH3_2_PDZ_4_, and GBD_1_SH3_4_PDZ_4_] and their corresponding partners have been identified (Pawson, 2007). The PDZ domain (also known as GLGF repeats or DHR domains) is typically an essential component of multi-domain scaffold proteins involved in cell polarity and intercellular interactions (Fanning and Anderson, 1996). It can selectively recognize the C-terminal peptide sequences on its partner protein and then assemble them into a complex and target specific subcellular localization sites (Tonikian et al., 2008). Based on this protein-peptide interaction, Gao et al. (2014) proposed a scaffold-free self-assembly strategy. This strategy was successfully demonstrated using the NAD (H) cycle system with L-*tert*-leucine as a model, achieving scaffold-free self-assembly technology. They fused the PDZ (PSD95/Dlg1/zo-1) domain and corresponding ligands (PDZlig) from metazoan cells separately with the octameric leucine dehydrogenase (LDH, derived from *Bacillus subtilis* BEST7613) and the dimeric formate dehydrogenase (FDH, derived from *Lodderomyces elongisporus* NRRL YB4239) (Figure 1A). The fusion proteins self-assembled into extended supramolecular interaction networks, significantly enhancing the efficiency and structural stability of the coenzyme cycling system involving NAD (H). Compared to their non-assembled counterparts, they exhibited better performance (Gao et al., 2014).

The SRC Homology 3 Domain (or SH3 domain) is a small protein domain containing 60 amino acid residues that are folded into beta-barrels with five or six β-strands arranged as two tightly packed anti-parallel β sheets (Schlessinger, 1994). It typically binds to proline-rich peptides in its respective binding partner. A SH3-ligand interaction strategy was used to successfully assemble methanol dehydrogenase (Mdh), 3-hexulose-6-phosphate synthase (Hps), and 6-phospho-3-hexuloseisomerase (Phi) into highly efficient enzyme complexes, significantly improving the conversion efficiency of methanol to fructose-6-phosphate (F6P) (Price et al., 2016). Meanwhile, in *Escherichia coli*, lactate dehydrogenase was utilized as an NADH scavenger to establish an “NADH sink.” By combining these two strategies, a 97-fold increase in extracellular F6P production and a 9-fold improvement in intracellular methanol consumption were successfully achieved (Price et al., 2016).

The GTPase binding domain (GBD) from the actin polymerization switch N-WASP could be recognized by the GTP-bound Cdc42. Dueber et al. (2009), used the GTPase binding domain (GBD), the SH3 domain, and the PDZ domain to build a synthetic scaffold to provide modular control over metabolic pathway flux. By varying the numbers of these three domains (GBD_x_SH3_y_PDZ_z_; x, y, z = number of domain repeats) to control the co-localization ratio of the interacting catalytic enzymes (e.g., atoB, HMGS, and HMGR), the optimal scaffold quantity is GBD_1_SH3_2_PDZ_2,_ and this engineering strategy led to a 77-fold increase in malic acid production (Dueber et al., 2009). By constructing a self-assembly enzyme reactor in *E. coli*, the stoichiometric ratio of two enzymes in the baicalein synthesis pathway was regulated to form an enzyme complex. This strategy significantly increased the titers of baicalein and scutellarein by 6.6 and 1.4 folds, respectively (Ji et al., 2021). Wei et al. utilized *tobacco mosaic virus* (TMV) virus-like particle (VLP) as a protein scaffold and orthogonal reactive protein pairs (SpyCatcher/SpyTag and SnoopCatcher/SnoopTag) as a linking module to assemble terpene biosynthesis in *E. coli*, enabling the production of amorpha-4,11-diene (Wei et al., 2020).

### 2.2 Peptide–peptide pair

In nature, there are numerous examples of optimizing metabolic pathway performance by forming multienzyme complexes. A prominent example is polyketide synthases (PKSs), which are considered among the most intricate proteins in nature. PKSs are classified into types I, II, and III and are involved in the synthesis of numerous compounds (Nivina et al., 2019). Through the sequential action of multiple catalytic modules, type I modular polyketide synthases are capable of extending, modifying, and terminating polyketide peptide chains. These interrelated modules interact with each other through docking domains (DDs) mediated by folding regions at the C- and N-termini (Weissman, 2016). Sun et al. (2022) utilized the DDs of type I *cis*-AT-PKS as mediators to develop a multi-enzyme assembly strategy named mimic PKS enzyme assembly line (mPKSeal), which mimics the assembly line of PKS enzymes (Figure 1B). This strategy was applied in engineered *E*. *coli* to enhance astaxanthin production and possesses the ability to co-locate enzymes within the cell, enabling the assembly of two or three enzyme units in different cellular environments (Sun et al., 2022). Their research also found that DDs from different PKSs but located on the same molecular evolutionary tree also possess enzyme assembly activity. The mPKSeal enzyme assembly strategy has tremendous potential for enhancing the efficiency of biocatalytic reactions by regulating the spatial positioning of enzymes without altering their abundance. These short-chain DDs have little significant impact on the catalytic activity of most enzyme assemblies, but they have a more pronounced effect on certain specific membrane proteins. Thus, this is an issue that needs to be noted when dealing with membrane proteins (Sun et al., 2022).

Kang et al. (2019) developed a scaffold-free modular enzyme assembly, which incorporated short peptide tags RIDD and RIAD derived from cAMP-dependent protein kinase (PKA) (Wong and Scott, 2004) and the A kinase-anchoring proteins (AKAPs) (Sarma et al., 2010), respectively. In *E. coli*, researchers successfully assembled enzyme complexes by combining the interaction peptides of RIAD and RIDD with the isopentenyl diphosphate isomerase (IDI) and Geranylgeranyl diphosphate synthase (CrtE), involved in the carotenoid biosynthesis pathway. This led to a significant increase in the production of carotenoids. Furthermore, in *S*. *cerevisiae*, the assembly of these two short peptides with the IDI and CrtE for the biosynthesis of lycopene resulted in a 58% increase in lycopene production (Kang et al., 2019). Xu et al. (2022) assembled two cytochrome P450 enzymes, ent-kaurene oxidase (KO) and kaurenoic acid 13*α*-hydroxylase (KAH), using RIAD and RIDD, successfully increasing the production of rubusoside and rebaudiosides in yeast. Fink et al. (2020), through orthogonally designed coiled-coil interaction domains, cluster resveratrol biosynthetic pathway enzymes, thereby increasing the yield of resveratrol in *E*. *coli*. The yield of resveratrol produced by this method is higher than that of direct enzyme fusion and internal protein-mediated fusion. At the same time, the biosynthesis of mevalonate in yeast was improved by this clustering method.

### 2.3 Protein-protein pair

Protein-protein pairs of special peptides in TatB/TatC can spontaneously interact to form aggregates. Henriques de Jesus et al. (2017) realized the co-localization of enzymes by exchanging the membrane anchors of the dhurrin biosynthesis pathway enzymes into TatB and TatC components of the twin-arginine translocation pathway with self-assembly properties. This method achieved a 4-fold increase in dhurrin titer and reduced the amount of intermediates and side products. CipA and CipB are two small proteins that form protein crystalline inclusions (PCIs) in the cytoplasm of *Photorhabdus luminescens*. Wang Y. et al. (2017) used CipA as a protein scaffold to bring together multiple enzymes (Vio enzymes) of the violacein biosynthetic pathway to explore its application *in vivo*. They found that the violacein production in the complex was significantly increased with fewer side-products (Wang Y. et al., 2017). More recently, Park et al. (2022) applied CipB scaffold proteins to bring P450s and reductase in close proximity, facilitating electron transfer between them. The development of strains producing lutein, apigenin, (+)-nootkatone, and L-3, 4-dihydroxyphenylalanine (l-DOPA) in *E. coli* has demonstrated the universal applicability of this electronic channel strategy. By using an implicit negative design, Sahtoe et al. (2022) generated beta sheet-mediated heterodimers capable of assembling into a variety of complexes. Their implicit negative design principle makes it possible to design higher-order asymmetric polyprotein complexes by rigid fusion of components through structured helical linkers. Moreover, due to the small size of the unfused protomers, the complex can be functionalized by easily fusing with the protein of interest by subunits (Sahtoe et al., 2022).

The scaffold assembly can realize the orderly arrangement of multiple enzymes, shorten the spatial distance of enzymes, accelerate sequential catalysis, and achieve a high yield. The composite formed by the scaffold assembly strategy can significantly improve the efficiency of enzyme catalysis in the biosynthesis of natural products and has broad application prospects in the fields of metabolic engineering and synthetic biology. However, until now, only four enzymes could be assembled sequentially. The reason is that the assembly of the enzyme requires the fusion expression of the scaffold and the enzyme through the joint, and misfolding is easy to occur during the fusion process, which will affect the assembly performance. On the other hand, the assembly of multiple enzymes is affected by steric hindrance, making it difficult to achieve sequential arrangement (Chen et al., 2023).

## 3 Nucleic acid scaffold

### 3.1 DNA scaffold

In addition to using protein scaffold approaches, the DNA double helix can serve as an alternative scaffold system. Compared with protein scaffolds, nucleic acid scaffolds have higher flexibility and maneuverability. With the advancement of gene editing technology, several molecular tools are available for efficient and specific DNA targeting, such as zinc finger proteins (ZFPs), transcription activator-like effector (TALE) proteins, and CRISPR-Cas (Kim and Kim, 2014). Moreover, the plasmid DNA as a configurable, stable, and robust scaffold for arranging biosynthetic enzymes in the cytoplasm is proposed.

#### 3.1.1 DNA scaffold based on zinc finger protein

In *E. coli*, the plasmid DNAs equipped with corresponding zinc finger protein binding sites were designed to assemble three different biosynthetic pathways to produce resveratrol, 1,2-propanediol, or mevalonate (Conrado et al., 2012). By varying the enzymatic ratios and the base pairs between each enzyme, the catalytic efficiency is improved, which leads to better production of final products. This similar approach had been applied to using zinc finger proteins (ZFPs) as adaptors to anchor the L-threonine biosynthetic genes. By using DNA scaffold assembly, the accumulation of the intermediate homoserine is reduced and significantly increases the efficiency of L-threonine biosynthesis due to the shortening of the distance between enzymes and the enhancement of the local concentration of metabolic products (Lee et al., 2013). In addition, Rahmana et al. (2014) utilized fusion proteins of chimeric acyl-ACP reductase (AAR) and aldehyde decarbonylase (ADO), or zinc finger proteins, as guides to assemble ADO/AAR with DNA scaffolds. The strain containing the fusion protein ADO-AAR showed a 4.8-fold increase in the production of branched alkanes. On the DNA scaffold, when the stoichiometric ratio of ADO to AAR was 3:1, the strain exhibited an 8.8-fold increase in production, reaching the optimal level of branched alkane synthesis (Rahmana et al., 2014). Liu et al. (2014) constructed a *B. subtilis* strain capable of producing *N*-acetylglucosamine (GlcNAc) and, for the first time, utilized DNA scaffolds to regulate the activities of glucosamine-6-phosphate synthase and GlcNAc-6-phosphate *N*-acetyltransferase, resulting in a GlcNAc titer of 4.55 g/L.

#### 3.1.2 DNA scaffold based on TALEs

**FIGURE 2:**
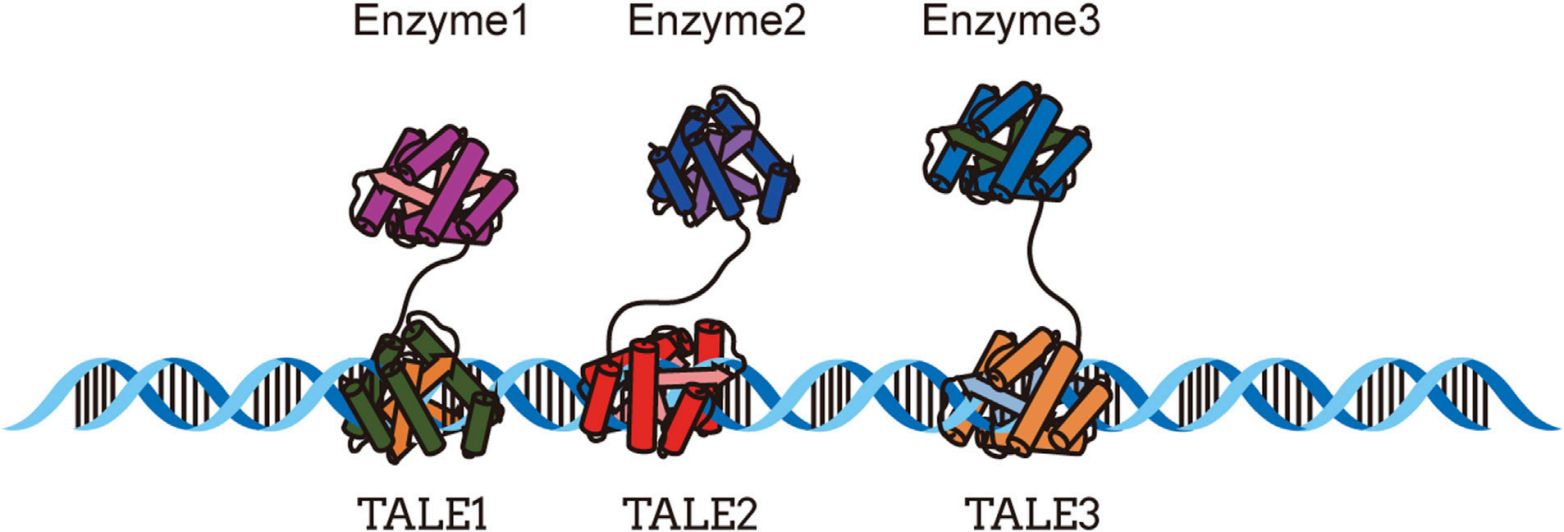
DNA scaffold system based on TALE, adapted from (Xie et al., 2019).

TALEs (transcription activator-like effectors) are effectors from the family III in *Xanthomonas* bacteria (Boch and Bonas, 2010), and different TALEs share similar structural domains. These domains are capable of binding to the host cell genome and act as transcription factors to recognize specific DNA repeat sequences (Boch et al., 2009). Based on the binding characteristics of TALEs to DNA, Zhu et al. (2016) developed a TALE-based DNA scaffold system and applied it to the biosynthesis of indole-3-acetic acid (IAA). Furthermore, in a modified TALE-DNA scaffold system, three fusion enzymes were successfully assembled in *E*. *coli* and significantly increased the production of a mevalonate-producing tri-enzymatic pathway (Xie et al., 2019) (Figure 2).

#### 3.1.3 DNA scaffold based on CRISPR-Cas

CRISPR-associated (Cas) nucleases are a class of DNA-binding proteins distinct from zinc finger enzymes and transcriptional activator-like effector proteins. Through the guidance of RNA molecules complementary to DNA sequences, the CRISPR-Cas DNA scaffold achieves Cas-specific customization (Lim et al., 2020). The Cas9 protein is ideally suited for modular enzyme assembly, with a high affinity for DNA and the ability to bind to specific DNA sequences (Berckman and Chen, 2019). By combining the dCas9 nuclease (from *Streptococcus pyogenes*) with the Spycatcher-Spytag chemical binding system, successful modular assembly of five enzymatic pathways involved in violacein biosynthesis was achieved, resulting in a significant increase in violacein production (Lim et al., 2020). In another study, two orthogonal SpyCatcher and SnoopCatcher pairs were bioconjugated onto two different dCas9 proteins, enabling them to guide the enzyme assembly to the DNA scaffold, resulting in a 2.8-fold increase in reducing sugar production compared to the unassembled enzyme (Berckman and Chen, 2020).

#### 3.1.4 DNA scaffold based on PCNA

Proliferating cell nuclear antigen (PCNA) is a trimeric ring-shaped protein (Moldovan et al., 2007) that binds to DNA as a scaffold for DNA-related enzymes. The fusion protein between the PCNA and the functional protein can act as a nanoscale part and self-assemble to form a functional nanohybrid complex. Fusion of three PCNA proteins with bacterial cytochrome P450 or one of the two electron transfer-related proteins can form a stable heterotrimer complex, resulting in increased local ferridoxin concentrations of P450 and ferredoxin reductase and high catalytic activity of electron transfer within the complex (Hirakawa and Nagamune, 2010).

### 3.2 RNA scaffold

By binding to the adapter, the RNA scaffold achieves highly specific binding to the target enzyme. Delebecque et al. (2011) designed and assembled multidimensional RNA structures to spatially organize proteins in cells and utilized this RNA scaffold to optimize a biosynthetic pathway for hydrogen production. A fluorescent protein library containing 8 aptamers and corresponding RNA domains was successfully assembled by fusing the active viral scaffold made of RNA with engineered proteins and specific RNA domains. The scaffold enables the co-localization of fragmented green fluorescent proteins to achieve precise measurement of cellular activity (Sachdeva et al., 2014). The application of this RNA scaffold to the synthesis pathway enzymes of pentadecane and succinic acid demonstrated the assembly of 0D, 1D, and 2D scaffolds. In the biosynthesis pathway of pentadecane, the 2D-assembled scaffold resulted in a 2.4-fold increase in pentadecane production (Sachdeva et al., 2014).

CRISPR-associated RNA scaffolds provide a powerful approach to the construction of synthetic gene programs. By inducing the expression of the dCas9 protein, we can achieve gene activation and inhibition, thereby enabling the directed expression of complex branching metabolic pathways (Zalatan et al., 2015). Pothoulakis et al. (2022) developed an RNA design approach for RNA origami scaffolds (termed sgRNAO) by recruiting activation domains from fused single-guide RNAs and RNA origami scaffolds to control gene expression in yeast. They successfully applied sgRNAOs to regulate the expression of enzymes involved in the violacein biosynthetic pathway (Pothoulakis et al., 2022).

Compared to DNA or protein-based scaffolds, RNA scaffolds, as non-coding synthetic scaffolds, offer greater flexibility. They can control protein spatial organization, such as distances and orientations between bound proteins, chemical dosage, and complex sizes, among others (Delebecque et al., 2012). However, although RNA scaffolds have certain advantages, they also have obvious disadvantages, including high synthesis costs, easy hydrolysis by nucleases, large environmental factors, and structural instability, which limit their application. Therefore, it requires further research and development to resolve these drawbacks (Geraldi et al., 2021).

## 4 Cellular scaffolds

### 4.1 Natural cellular scaffolds

**FIGURE 3:**
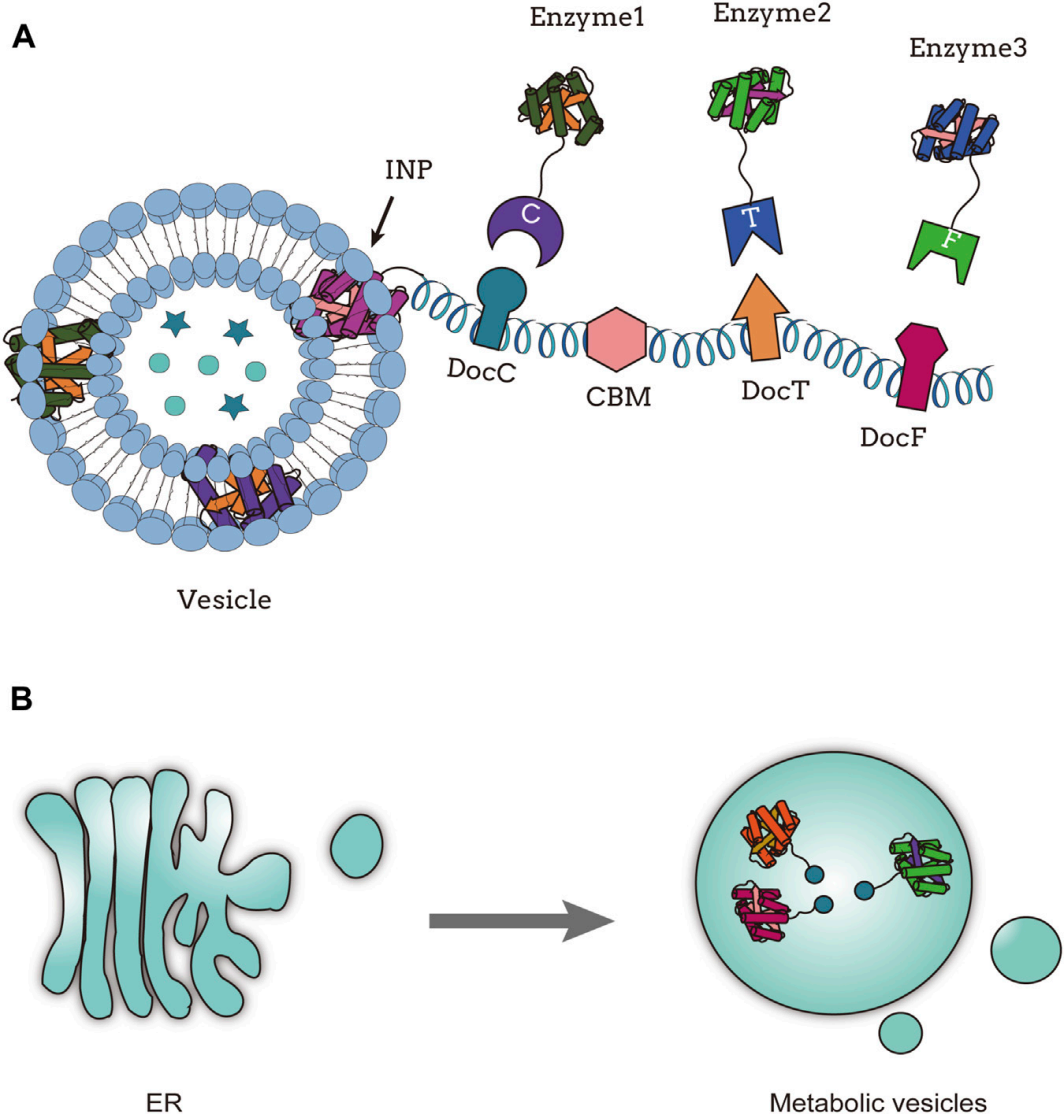
Organelle scaffold; **(A)** Functional assembly of multienzyme systems on outer membrane vesicles, adapted from (Park et al., 2014); **(B)** Compartmental assembly of endoplasmic reticulum derived metabolic vesicles, adapted from (Reifenrath et al., 2020).

As subcellular structures within cells, the integrity and autonomy of organelles have sparked scientists interest in using them as scaffolds for enzyme assembly (Liu et al., 2023). Bacterial microcompartments (BMCs) (Kerfeld et al., 2018) are self-assembling organelles composed of enzymatic cores that participate in the metabolism of various organic compounds such as 1,2-propanediol (Petit et al., 2013), ethanolamine (Petit et al., 2013), fucose, and rhamnose (Parsons et al., 2008), playing a crucial role in carbon fixation processes (Lawrence et al., 2014). In *E*. *coli*, reconstitution of recombinant microcompartments can be achieved by translocating the entire propanediol utilization (Pdu) operon from *Citrobacter freundii* (Parsons et al., 2008). The *Zymomonas mobilis* enzymes pyruvate decarboxylase (Pdc) and alcohol dehydrogenase (Adh) can be targeted to PduP of *C. freundii* to form a simple ethanol bioreactor inside the Pdu microcompartment shell (Lawrence et al., 2014). The enzymes Pdc and Adh, necessary for ethanol production, are expressed heterologously using a foreign host and targeted to the protein shell. In strains containing target enzymes, the ethanol yield significantly increases when the protein shell content is highest, including the strains producing shell proteins P18-Pdc and D18-Adh (Lawrence et al., 2014). In another study, the known Pdu (D18 and P18) targeting peptides were fused with four different 1,2-propanediol synthetic enzymes to create fusion proteins that target the empty Pdu BMC system. The fusion strategy of targeting peptides with all proteins involved in 1,2-propanediol synthesis significantly increased the product yield (Lee et al., 2016). Studies have shown that BMCs have great potential for constructing organelle scaffolds and are relatively easy to design, especially when it comes to the metabolism of toxic intermediates (Lawrence et al., 2014). Nielsen et al. (2013) transferred the biosynthetic pathway of the aromatic defense compound dhurrin [D-glucopyranosyloxy-(S)-p-hydroxymandelonitrile, a cyanogenic glucoside] to plant chloroplasts, utilizing photo-induced water splitting as the electron source to drive product synthesis in a light-dependent manner. The biosynthetic pathway of dhurrin involves three ER-localized enzymes, including two P450 enzymes, CYP79A1 and CYP71E1, as well as an NADPH cytochrome P450 oxidoreductase, POR. The chloroplast stroma provides a reducing environment for P450 enzymes, thereby enhancing their stability (Nielsen et al., 2013). Gram-negative bacteria release spherical nanoscale particles called outer membrane vesicles (OMVs) during their growth process. These vesicles have a composition similar to the bacterial outer membrane, containing lipopolysaccharides (LPSs), outer membrane proteins (OMPs), and phospholipids (Beveridge, 1999). In order to achieve the goal of hijacking the bacterial cell export pathway to simultaneously produce, package, and release an active enzyme, phosphotriesterase (PTE), Alves et al. (2015) attempted to establish synthetic linkages between enzymes and proteins known to exist in the outer membrane. They used the SpyCatcher/SpyTag (SC/ST) bioconjugated system to connect OmpA proteins present in OMVs to phosphotriesterase from *Brevundimonas diminuta*. A PTE-SpyCatcher (PTE-SC) fusion protein and a SpyTag transmembrane porin protein (OmpA-ST) were constructed. The coexpression of OmpA-ST with PTE-SC not only reduced the toxicity of PTE and improved the overall PTE production level, but also enhanced the stability of packaging enzymes against repeated freeze-thaw cycles (Alves et al., 2015). By employing a truncated ice nucleation protein anchoring motif (INP) on OMVs, a trivalent protein scaffold containing three divergent cohesin domains was utilized for site-specific expression of a three-enzyme cascade, resulting in a 23-fold increase in glucose production (Park et al., 2014) (Figure 3A). Yang et al. (2021) developed a metabolically engineered strain of *E. coli* to produce seven natural colorants, and they significantly increased the yield of seven natural colorants through cell morphological engineering, IMV and OMV formation, and fermentation optimization strategies. *S*. *cerevisiae* possesses various subcellular compartments, making it an ideal host for building heterologous natural product biosynthesis. Shi et al. (2021) used the PLN1 protein to target endoplasmic reticulum-localized cytochrome P450 enzymes and protopanaxadiol (PPD) synthase (PPDs) towards lipid droplets [DDs, the storage organelle for dammarenediol-II (DD)], resulting in a 394% increase in the conversion of DD to PPD and an elevated conversion rate of DD to 86.0%. Peroxisomes are organelles involved in fatty acid degradation. In yeast, significant improvements in the production of fatty acid derivatives, such as fatty alcohols, alkanes, and olefins, can be achieved by engineering peroxisomes. For example, increasing the number of peroxisomes can triple the production of fatty acid derivatives (Zhou et al., 2016).

### 4.2 Synthetic organelle scaffold

Cellular organelle scaffolds can be artificially designed to meet various metabolic pathway requirements. Lipids are widely present in cells and can form cell membranes, with many proteins anchored to these membrane structures. Inspired by this, researchers have attempted to use lipids as synthetic scaffolds to achieve co-assembly of lipids and target proteins. They discovered that, unlike most bacteriophages, bacteriophage *ϕ*6 contains a protein nucleocapsid surrounded by a lipid envelope and several membrane proteins (Sinclair et al., 1975). During infection of the natural host (Stitt and Mindich, 1983) and in strains of *E*. *coli* expressing genes encoding bacteriophages *ϕ*6 viral proteins (Johnson and Mindich, 1994), assembly intermediates of bacteriophage *ϕ*6 with lipid-like structures were found. When only genes encoding the three viral proteins P8, P9, and P12 are expressed in *E*. *coli*, circular particles composed of a mixture of lipids and proteins can be observed by cryo-electron microscopy (Sarin et al., 2012). Myhrvold et al. (2016) engineered synthetic lipid-containing scaffolds (SLSs) in *E*. *coli*. The scaffold consists of the membrane protein P9 and the non-structural protein P12, which are required for the formation of the particle structure. The target proteins are fused to the C-terminus of P9 to position them on the scaffold. TnaA and FMO enzymes involved in indigo biosynthesis were fused to the C-terminus of P9 to construct lipid scaffolds for increased indigo production (Myhrvold et al., 2016). By utilizing a protein scaffold based on the plant lipid droplet protein oleosin and cohesin-dockerin interaction pairs recruited upstream enzymes, the final three reaction steps of yeast ester biosynthesis were assembled on lipid droplets (LDs) within yeast cells. This resulted in a doubling of the synthesis rate of ethyl acetate (Lin et al., 2017). There are derived vesicles containing metabolic pathway enzymes in the endoplasmic reticulum (“metabolic vesicles”). Reifenrath et al. (2020) integrate the three enzymes involved in the production of *cis,cis*-muconic acid into yeast vesicles, construct an ER-derived synthetic cell envelope containing the metabolic pathway, and map the yeast metabolism (Figure 3B). Wei et al. (2022) used *β*-Cav1 caveolar vesicles as an enzyme assembly scaffold to immobilize enzymes involved in the biosynthesis of *α*-farnesene from isopentenyl diphosphate and dimethylallyl pyrophosphate through non-covalent interactions or covalent protein reactions on the *β*-Cav1 caveolar vesicles. They successfully constructed a multienzyme complex called multi-enzyme caveolar membranes (MCMs), which increased the catalytic efficiency of *α*-farnesene by 10-fold compared to the non-assembled enzymes (Wei et al., 2022).

## 5 Discussion and summary

With the advancement of biotechnology, building synthetic pathways into heterologous microbial hosts became possible (Jiang et al., 2021). However, this none-native expression of catalytic enzymes still faces numerous issues and challenges, including low productivity and yield, rapid diffusion and degradation of key intermediates, and the accumulation of toxic metabolites (Jiang et al., 2021). To address these issues, scientists have been organizing these enzymes into molecular complexes in space to enhance the local concentration of enzymes and metabolites, thereby improving reaction flux (Wang S. Z. et al., 2017). Currently, various assembly strategies have been derived based on the interactions of biomacromolecules such as nucleic acids, peptides, and scaffold proteins (Table 1). In comparison, the spatial organization of biosynthetic pathway enzymes through synthetic scaffolds has proven to be an effective method for enhancing reaction rates and biosynthetic yields while also improving host viability. Despite achieving a certain level of success, the precise prediction of artificial scaffold assembly remains challenging due to the complexity of enzyme structures. Furthermore, the catalytic efficiency of multi-enzyme complexes is influenced by numerous parameters, and the impact mechanism of linker sequences on both the multi-enzyme complex and substrate channeling effects remains undisclosed. Therefore, further research is needed to address the factors in the design-built-test-learn (DBTL) cycles of establishing artificial scaffold systems.

**TABLE 1:** Examples and overview of artificial scaffold systems.

Types of scaffolds	Description	Host	Application	References
Protein scaffold	PDZ and PDZ ligand	*E.coli*; *Pichia pastoris*	Enhance the biosynthesis yield of baicalein and scutellarein; facilitate the biosynthesis of ginsenoside precursors; Improve the production of itaconic acid	Zhao et al. (2016), Yang et al. (2017), Ji et al. (2021)
	SH3-ligand interaction pair	*E.coli*	Drive the conversion of methanol into H6P; promote the production of malic acid	Price et al. (2016), Somasundaram et al. (2020)
	PduA*-Multi-Enzyme Complex System	*E.coli*	Improve the synthesis efficiency of 5-aminolevulinic acid (5-ALA)	Luo et al. (2022)
	Cohesin-dockerin (Coh-Doc) pair	*S. cerevisiae*	Enhance the production rate of NADH; improve the metabolic flux of pyruvate	Liu et al. (2013), Kim et al. (2016)
	PDZ and SH3 domains	*E.coli*	Increase the effective concentration of myoinositol	Moon et al. (2010)
	RIAD and RIDD short peptide tags	*E.Coli; S. cerevisiae*	Increase carotenoid production by 5.7-fold and lycopene production by 58%; increase the yield of rubusosides and rebaudiosides	Kang et al. (2019), Xu et al. (2022)
	Artificial Protein Scaffold System (AProSS)	*S. cerevisiae*	The yield of violacein and deoxyviolacein increased by 29% and 63%, respectively, while the ratio of violacein to deoxyviolacein increased by 18%	Li et al. (2018)
	GBD, SH3, and PDZ domain	*E.coli*	Increase the production of indigoidine, gamma-aminobutyric acid, butyrate, and R-(−)-linalool; increase the production of methylhydroxybutyrate by 77 yields	Dueber et al. (2009), Baek et al. (2013), Pham et al. (2015), Pham et al. (2016), Wang et al. (2020), Wu et al. (2021)
	SpyCatcher/SpyTag and SnoopCatcher/SnoopTag pairs	*E.coli*	Increase the biosynthetic flux of carotenoids	Qu et al. (2019)
	Tobacco mosaic virus (TMV) virus-like particle (VLP), SpyCatcher/SpyTag and SnoopCatcher/SnoopTag	*E.coli*	Realization of the production of amorpha-4,11-diene	Wei et al. (2020)
	mimic PKS enzyme assembly line (mPKSeal)	*E.coli*	Improve the production of astaxanthin	Sun et al. (2022)
Nucleic acid scaffold	ADB1, ADB2, and ADB3	*Bacillus subtilis*	Increase the production of *N*-acetylglucosamine; enhance the biosynthesis of *L*-threonine	Lee et al. (2013), Liu et al. (2014)
	ADO and AAR	*E.coli*	Enhance the production potential of linear n-alkanes	Rahmana et al. (2014)
	TALEs	*E.coli*	Increase the biosynthesis of indole-3-acetic acid (IAA)	Zhu et al. (2016), Xie et al. (2019)
	ZF domains	*E.coli*	Enhance the metabolism of resveratrol, 1,2-propanediol, and mevalonate	Conrado et al. (2012)
	PCNA	*E.coli*	Increase the catalytic activity of P450 and electron transfer-associated proteins	Hirakawa and Nagamune (2010)
	dCas9, SpyCatcher, and SnoopCatcher pairs	*E.coli*	Increase the yield of reducing sugars by 2.8 folds	Berckman and Chen (2020)
	dCas9, MS2 and PP7 aptamers	*S. cerevisiae*	Regulate the expression of enzymes involved in the violacein biosynthetic pathway to control metabolic flux	Pothoulakis et al. (2022)
	2DRNA scaffolds	*E.coli*	Increase the metabolic output of the pathway for pentadecane production	Delebecque et al. (2011), Sachdeva et al. (2014)
	RNA Scaffold, MS2 and PP7 aptamers	*E.coli*	The fluorescence intensity in the GFP cleavage assay increased by 2.25-fold, while the multi-enzyme efficiency in the IAA synthesis pathway increased by 1.43-fold	Team and Chen (2015)
	CRISPR scRNA	*S. cerevisiae*	Redirecting metabolic flux in a complex branched metabolic pathway	Zalatan et al. (2015)
Organelle scaffold	MCMs	*E.coli*	Improving the production of *α*-farnesene	Wei et al. (2022)
	ER-Derived Vesicles	*S. cerevisiae*	Constructing a *cis*,*cis*-muconic acid (CCM) biosynthetic pathway in vesicles to assess its feasibility	Reifenrath et al. (2020)
	lipid droplets (LDs)	*S. cerevisiae*	The production rate of ethyl acetate has been increased by nearly two-fold	Lin et al. (2017)
	outer membrane vesicles (OMVs)	*E.coli*	The glucose yield has increased by 23-fold compared to the free enzyme	Park et al. (2014)
	protein cages	*E.coli*	The production of lycopene has increased by 8.5-fold	Kang et al. (2022)

The computational inference of biological systems has emerged as a transformative field, driven by the confluence of advanced computational methods, machine learning techniques, and ever-expanding biological data. The best-known case is protein structure prediction, and it has been a grand challenge for decades. Since the advancement of the computational implication of a protein’s spatial arrangement of atoms and computing power, several artificial intelligence (AI) systems have been successfully applied to predict the structure of protein complexes (e.g., the AlphaFold-Multimer, RoseTTAFolds, and trRosetta) (Baek et al., 2021; Du et al., 2021; Brems et al., 2022; Ivanov et al., 2022). In addition, the use of artificial intelligence computational methods can effectively find scaffold proteins from protein interactors and fully reveal their functions (Oh and Yi, 2016). Currently, we may *de novo* design protein structures for specific purposes (Watson et al., 2023).

In contrast to protein-based scaffold systems, using nucleic acid as a scaffold provides its unique advantages. The DNA scaffold systems are more stable, easier to design, and have higher stability (Chen et al., 2014; Siu et al., 2015). However, the DNA scaffolds also serve several drawbacks, such as high cost and high error rates in self-assembly, making the folding process in living cells susceptible to environmental factors such as temperature and ions and difficult to manipulate. In addition, the DNA topology is single in dimension and has the problem of supercoiling (Polka et al., 2016). DNA origami is a technology for designing complex three-dimensional DNA structures. This has been broadly applied in nanotechnology, creating nanoscale structures for various purposes, including drug delivery and molecular computing (Zhou et al., 2023). Thus, DNA origami could greatly assist in resolving the issues and unlocking the great potential of using DNA scaffolds in metabolic engineering. Alternatively, the RNA can also be used as a scaffold for the self-assembly of multi-enzyme complexes. However, its discrete structure prevents the formation of complex geometric shapes in living organisms, limiting its application compared to other types of scaffolds (Lv et al., 2020). AI models also open up new opportunities for predicting the secondary and tertiary structures of RNA molecules, providing insights into designing new functions and interactions and improving stability (Sato and Hamada, 2023).

Cellular scaffolds constructed based on different organelles or membranes have achieved varying degrees of success. However, due to the levels of complexity of biological parts (e.g., membranes, proteins, and protein interactions), the working mechanism of such scaffold systems still needs to be further explored. Synthetic biology aims to combine multidisciplinary disciplines that pursue the development new biological parts or systems. The promise of using AI technology to resolve complex biological questions is now is gradually revealed. Combining these two technologies, we can better understand the mechanisms of different types of artificial scaffolds and design streamlined artificial scaffolds that provide a broader range of engineering applications.

## References

[B1] Alves N. J. Turner K. B. Daniele M. A. Oh E. Medintz I. L. Walper S. A. (2015). Bacterial nanobioreactors--directing enzyme packaging into bacterial outer membrane vesicles. ACS Appl. Mater Interfaces 7 (44), 24963–24972. 10.1021/acsami.5b08811 26479678

[B2] Artzi L. Bayer E. A. Moraïs S. (2017). Cellulosomes: bacterial nanomachines for dismantling plant polysaccharides. Nat. Rev. Microbiol. 15 (2), 83–95. 10.1038/nrmicro.2016.164 27941816

[B3] Baek J. M. Mazumdar S. Lee S. W. Jung M. Y. Lim J. H. Seo S. W. (2013). Butyrate production in engineered *Escherichia coli* with synthetic scaffolds. Biotechnol. Bioeng. 110 (10), 2790–2794. 10.1002/bit.24925 23568786

[B4] Baek M. DiMaio F. Anishchenko I. Dauparas J. Ovchinnikov S. Lee G. R. (2021). Accurate prediction of protein structures and interactions using a three-track neural network. Science 373 (6557), 871–876. 10.1126/science.abj8754 34282049 PMC7612213

[B5] Berckman E. A. Chen W. (2019). Exploiting dCas9 fusion proteins for dynamic assembly of synthetic metabolons. Chem. Commun. (Camb) 55 (57), 8219–8222. 10.1039/c9cc04002a 31210215 PMC7725109

[B6] Berckman E. A. Chen W. (2020). A modular approach for dCas9-mediated enzyme cascading via orthogonal bioconjugation. Chem. Commun. (Camb) 56 (77), 11426–11428. 10.1039/d0cc04196c 32840530

[B7] Beveridge T. J. (1999). Structures of gram-negative cell walls and their derived membrane vesicles. J. Bacteriol. 181 (16), 4725–4733. 10.1128/jb.181.16.4725-4733.1999 10438737 PMC93954

[B8] Boch J. Bonas U. (2010). Xanthomonas AvrBs3 family-type III effectors: discovery and function. Annu. Rev. Phytopathol. 48, 419–436. 10.1146/annurev-phyto-080508-081936 19400638

[B9] Boch J. Scholze H. Schornack S. Landgraf A. Hahn S. Kay S. (2009). Breaking the code of DNA binding specificity of TAL-type III effectors. Science 326 (5959), 1509–1512. 10.1126/science.1178811 19933107

[B10] Brems M. A. Runkel R. Yeates T. O. Virnau P. (2022). AlphaFold predicts the most complex protein knot and composite protein knots. Protein Sci. 31 (8), e4380. 10.1002/pro.4380 35900026 PMC9278004

[B11] Castellana M. Wilson M. Z. Xu Y. Joshi P. Cristea I. M. Rabinowitz J. D. (2014). Enzyme clustering accelerates processing of intermediates through metabolic channeling. Nat. Biotechnol. 32 (10), 1011–1018. 10.1038/nbt.3018 25262299 PMC4666537

[B12] Chen R. Chen Q. Kim H. Siu K. H. Sun Q. Tsai S. L. (2014). Biomolecular scaffolds for enhanced signaling and catalytic efficiency. Curr. Opin. Biotechnol. 28, 59–68. 10.1016/j.copbio.2013.11.007 24832076

[B13] Chen Z. Wu T. Yu S. Li M. Fan X. Huo Y. X. (2023). Self-assembly systems to troubleshoot metabolic engineering challenges. Trends Biotechnol. 10.1016/j.tibtech.2023.06.009 37451946

[B14] Conrado R. J. Varner J. D. DeLisa M. P. (2008). Engineering the spatial organization of metabolic enzymes: mimicking nature's synergy. Curr. Opin. Biotechnol. 19 (5), 492–499. 10.1016/j.copbio.2008.07.006 18725290

[B15] Conrado R. J. Wu G. C. Boock J. T. Xu H. Chen S. Y. Lebar T. (2012). DNA-guided assembly of biosynthetic pathways promotes improved catalytic efficiency. Nucleic Acids Res. 40 (4), 1879–1889. 10.1093/nar/gkr888 22021385 PMC3287197

[B16] Delebecque C. J. Lindner A. B. Silver P. A. Aldaye F. A. (2011). Organization of intracellular reactions with rationally designed RNA assemblies. Science 333 (6041), 470–474. 10.1126/science.1206938 21700839

[B17] Delebecque C. J. Silver P. A. Lindner A. B. (2012). Designing and using RNA scaffolds to assemble proteins *in vivo* . Nat. Protoc. 7 (10), 1797–1807. 10.1038/nprot.2012.102 22955695

[B18] Du Z. Su H. Wang W. Ye L. Wei H. Peng Z. (2021). The trRosetta server for fast and accurate protein structure prediction. Nat. Protoc. 16 (12), 5634–5651. 10.1038/s41596-021-00628-9 34759384

[B19] Dueber J. E. Wu G. C. Malmirchegini G. R. Moon T. S. Petzold C. J. Ullal A. V. (2009). Synthetic protein scaffolds provide modular control over metabolic flux. Nat. Biotechnol. 27 (8), 753–759. 10.1038/nbt.1557 19648908

[B20] Fanning A. S. Anderson J. M. (1996). Protein-protein interactions: PDZ domain networks. Curr. Biol. 6 (11), 1385–1388. 10.1016/s0960-9822(96)00737-3 8939589

[B21] Fink T. Stevovic B. Verwaal R. Roubos J. A. Gaber R. Bencina M. (2020). Metabolic enzyme clustering by coiled coils improves the biosynthesis of resveratrol and mevalonate. Amb. Express 10 (1), 97. 10.1186/s13568-020-01031-5 32448937 PMC7246283

[B22] Gad S. Ayakar S. (2021). Protein scaffolds: a tool for multi-enzyme assembly. Biotechnol. Rep. (Amst) 32, e00670. 10.1016/j.btre.2021.e00670 34824995 PMC8605239

[B23] Gao X. Yang S. Zhao C. Ren Y. Wei D. (2014). Artificial multienzyme supramolecular device: highly ordered self-assembly of oligomeric enzymes *in vitro* and *in vivo* . Angew. Chem. Int. Ed. Engl. 53 (51), 14027–14030. 10.1002/anie.201405016 25319901

[B24] Geraldi A. Khairunnisa F. Farah N. Bui L. M. Rahman Z. (2021). Synthetic scaffold systems for increasing the efficiency of metabolic pathways in microorganisms. Biol. (Basel) 10 (3), 216. 10.3390/biology10030216 PMC7998396 33799683

[B25] Henriques de Jesus M. P. R. Zygadlo Nielsen A. Busck Mellor S. Matthes A. Burow M. Robinson C. (2017). Tat proteins as novel thylakoid membrane anchors organize a biosynthetic pathway in chloroplasts and increase product yield 5-fold. Metab. Eng. 44, 108–116. 10.1016/j.ymben.2017.09.014 28962875

[B26] Hirakawa H. Nagamune T. (2010). Molecular assembly of P450 with ferredoxin and ferredoxin reductase by fusion to PCNA. Chembiochem 11 (11), 1517–1520. 10.1002/cbic.201000226 20607777

[B27] Ivanov Y. D. Taldaev A. Lisitsa A. V. Ponomarenko E. A. Archakov A. I. (2022). Prediction of monomeric and dimeric structures of CYP102A1 using AlphaFold2 and AlphaFold multimer and assessment of point mutation effect on the efficiency of intra- and interprotein electron transfer. Molecules 27 (4), 1386. 10.3390/molecules27041386 35209175 PMC8874714

[B28] Ji D. Li J. Xu F. Ren Y. Wang Y. (2021). Improve the biosynthesis of baicalein and scutellarein via manufacturing self-assembly enzyme reactor *in vivo* . ACS Synth. Biol. 10 (5), 1087–1094. 10.1021/acssynbio.0c00606 33880917

[B29] Jiang Y. Zhang X. Yuan H. Huang D. Wang R. Liu H. (2021). Research progress and the biotechnological applications of multienzyme complex. Appl. Microbiol. Biotechnol. 105 (5), 1759–1777. 10.1007/s00253-021-11121-4 33564922

[B30] Johnson M. D. 3rd Mindich L. (1994). Plasmid-directed assembly of the lipid-containing membrane of bacteriophage phi 6. J. Bacteriol. 176 (13), 4124–4132. 10.1128/jb.176.13.4124-4132.1994 8021194 PMC205612

[B31] Kang W. Ma T. Liu M. Qu J. Liu Z. Zhang H. (2019). Modular enzyme assembly for enhanced cascade biocatalysis and metabolic flux. Nat. Commun. 10 (1), 4248. 10.1038/s41467-019-12247-w 31534134 PMC6751169

[B32] Kang W. Ma X. Kakarla D. Zhang H. Fang Y. Chen B. (2022). Organizing enzymes on self-assembled protein cages for cascade reactions. Angew. Chem. Int. Ed. Engl. 61 (52), e202214001. 10.1002/anie.202214001 36288455

[B33] Kerfeld C. A. Aussignargues C. Zarzycki J. Cai F. Sutter M. (2018). Bacterial microcompartments. Nat. Rev. Microbiol. 16 (5), 277–290. 10.1038/nrmicro.2018.10 29503457 PMC6022854

[B34] Kim H. Kim J. S. (2014). A guide to genome engineering with programmable nucleases. Nat. Rev. Genet. 15 (5), 321–334. 10.1038/nrg3686 24690881

[B35] Kim S. Bae S. J. Hahn J. S. (2016). Redirection of pyruvate flux toward desired metabolic pathways through substrate channeling between pyruvate kinase and pyruvate-converting enzymes in *Saccharomyces cerevisiae* . Sci. Rep. 6, 24145. 10.1038/srep24145 27052099 PMC4823786

[B36] Lawrence A. D. Frank S. Newnham S. Lee M. J. Brown I. R. Xue W. F. (2014). Solution structure of a bacterial microcompartment targeting peptide and its application in the construction of an ethanol bioreactor. ACS Synth. Biol. 3 (7), 454–465. 10.1021/sb4001118 24933391 PMC4880047

[B37] Lee J. H. Jung S. C. Bui le M. Kang K. H. Song J. J. Kim S. C. (2013). Improved production of L-threonine in *Escherichia coli* by use of a DNA scaffold system. Appl. Environ. Microbiol. 79 (3), 774–782. 10.1128/AEM.02578-12 23160128 PMC3568567

[B38] Lee M. J. Brown I. R. Juodeikis R. Frank S. Warren M. J. (2016). Employing bacterial microcompartment technology to engineer a shell-free enzyme-aggregate for enhanced 1,2-propanediol production in *Escherichia coli* . Metab. Eng. 36, 48–56. 10.1016/j.ymben.2016.02.007 26969252 PMC4909751

[B39] Li T. Chen X. Cai Y. Dai J. (2018). Artificial Protein Scaffold System (AProSS): an efficient method to optimize exogenous metabolic pathways in *Saccharomyces cerevisiae* . Metab. Eng. 49, 13–20. 10.1016/j.ymben.2018.07.006 30010058

[B40] Lim S. Kim J. Kim Y. Xu D. Clark D. S. (2020). CRISPR/Cas-directed programmable assembly of multi-enzyme complexes. Chem. Commun. (Camb) 56 (36), 4950–4953. 10.1039/d0cc01174f 32239050

[B41] Lin J. L. Zhu J. Wheeldon I. (2017). Synthetic protein scaffolds for biosynthetic pathway colocalization on lipid droplet membranes. ACS Synth. Biol. 6 (8), 1534–1544. 10.1021/acssynbio.7b00041 28497697

[B42] Liu F. Banta S. Chen W. (2013). Functional assembly of a multi-enzyme methanol oxidation cascade on a surface-displayed trifunctional scaffold for enhanced NADH production. Chem. Commun. (Camb) 49 (36), 3766–3768. 10.1039/c3cc40454d 23535691

[B43] Liu M. Wang Y. Jiang H. Han Y. Xia J. (2023). Synthetic multienzyme assemblies for natural product biosynthesis. Chembiochem 24 (6), e202200518. 10.1002/cbic.202200518 36625563

[B44] Liu Y. Zhu Y. Ma W. Shin H. D. Li J. Liu L. (2014). Spatial modulation of key pathway enzymes by DNA-guided scaffold system and respiration chain engineering for improved N-acetylglucosamine production by Bacillus subtilis. Metab. Eng. 24, 61–69. 10.1016/j.ymben.2014.04.004 24815549

[B45] Lumsden J. Coggins J. R. (1977). The subunit structure of the arom multienzyme complex of Neurospora crassa. A possible pentafunctional polypeptide chain. Biochem. J. 161 (3), 599–607. 10.1042/bj1610599 139889 PMC1164546

[B46] Luo Z. Pan F. Zhu Y. Du S. Yan Y. Wang R. (2022). Synergistic improvement of 5-aminolevulinic acid production with synthetic scaffolds and system pathway engineering. ACS Synth. Biol. 11 (8), 2766–2778. 10.1021/acssynbio.2c00157 35939037

[B47] Lv X. Cui S. Gu Y. Li J. Du G. Liu L. (2020). Enzyme assembly for compartmentalized metabolic flux control. Metabolites 10 (4), 125. 10.3390/metabo10040125 32224973 PMC7241084

[B48] Moldovan G. L. Pfander B. Jentsch S. (2007). PCNA, the maestro of the replication fork. Cell 129 (4), 665–679. 10.1016/j.cell.2007.05.003 17512402

[B49] Moon T. S. Dueber J. E. Shiue E. Prather K. L. (2010). Use of modular, synthetic scaffolds for improved production of glucaric acid in engineered *E. coli* . Metab. Eng. 12 (3), 298–305. 10.1016/j.ymben.2010.01.003 20117231

[B50] Myhrvold C. Polka J. K. Silver P. A. (2016). Synthetic lipid-containing scaffolds enhance production by colocalizing enzymes. ACS Synth. Biol. 5 (12), 1396–1403. 10.1021/acssynbio.6b00141 27487319

[B51] Nielsen A. Z. Ziersen B. Jensen K. Lassen L. M. Olsen C. E. Møller B. L. (2013). Redirecting photosynthetic reducing power toward bioactive natural product synthesis. ACS Synth. Biol. 2 (6), 308–315. 10.1021/sb300128r 23654276

[B52] Nivina A. Yuet K. P. Hsu J. Khosla C. (2019). Evolution and diversity of assembly-line polyketide synthases. Chem. Rev. 119 (24), 12524–12547. 10.1021/acs.chemrev.9b00525 31838842 PMC6935866

[B53] Oh K. Yi G. S. (2016). Prediction of scaffold proteins based on protein interaction and domain architectures. BMC Bioinforma., 17 (Suppl. 6), 220. 10.1186/s12859-016-1079-5 PMC4965726 27490120

[B54] Park M. Sun Q. Liu F. DeLisa M. P. Chen W. (2014). Positional assembly of enzymes on bacterial outer membrane vesicles for cascade reactions. PLoS One 9 (5), e97103. 10.1371/journal.pone.0097103 24820175 PMC4018249

[B55] Park S. Y. Eun H. Lee M. H. Lee S. Y. (2022). Metabolic engineering of *Escherichia coli* with electron channelling for the production of natural products. Nat. Catal. 5 (8), 726–737. 10.1038/s41929-022-00820-4

[B56] Parsons J. B. Dinesh S. D. Deery E. Leech H. K. Brindley A. A. Heldt D. (2008). Biochemical and structural insights into bacterial organelle form and biogenesis. J. Biol. Chem. 283 (21), 14366–14375. 10.1074/jbc.M709214200 18332146

[B57] Pawson T. (2007). Dynamic control of signaling by modular adaptor proteins. Curr. Opin. Cell Biol. 19 (2), 112–116. 10.1016/j.ceb.2007.02.013 17317137

[B58] Petit E. LaTouf W. G. Coppi M. V. Warnick T. A. Currie D. Romashko I. (2013). Involvement of a bacterial microcompartment in the metabolism of fucose and rhamnose by Clostridium phytofermentans. PLoS One 8 (1), e54337. 10.1371/journal.pone.0054337 23382892 PMC3557285

[B59] Pham V. D. Lee S. H. Park S. J. Hong S. H. (2015). Production of gamma-aminobutyric acid from glucose by introduction of synthetic scaffolds between isocitrate dehydrogenase, glutamate synthase and glutamate decarboxylase in recombinant *Escherichia coli* . J. Biotechnol. 207, 52–57. 10.1016/j.jbiotec.2015.04.028 25997833

[B60] Pham V. D. Somasundaram S. Lee S. H. Park S. J. Hong S. H. (2016). Redirection of metabolic flux into novel gamma-aminobutyric acid production pathway by introduction of synthetic scaffolds strategy in Escherichia coli. Appl. Biochem. Biotechnol. 178 (7), 1315–1324. 10.1007/s12010-015-1948-9 26667817

[B61] Polka J. K. Hays S. G. Silver P. A. (2016). Building spatial synthetic biology with compartments, scaffolds, and communities. Cold Spring Harb. Perspect. Biol. 8 (8), a024018. 10.1101/cshperspect.a024018 27270297 PMC4968161

[B62] Pothoulakis G. Nguyen M. T. A. Andersen E. S. (2022). Utilizing RNA origami scaffolds in *Saccharomyces cerevisiae* for dCas9-mediated transcriptional control. Nucleic Acids Res. 50 (12), 7176–7187. 10.1093/nar/gkac470 35648481 PMC9262615

[B63] Price J. V. Chen L. Whitaker W. B. Papoutsakis E. Chen W. (2016). Scaffoldless engineered enzyme assembly for enhanced methanol utilization. Proc. Natl. Acad. Sci. U. S. A. 113 (45), 12691–12696. 10.1073/pnas.1601797113 27791059 PMC5111641

[B64] Qu J. Cao S. Wei Q. Zhang H. Wang R. Kang W. (2019). Synthetic multienzyme complexes, catalytic nanomachineries for cascade biosynthesis *in vivo* . ACS Nano 13 (9), 9895–9906. 10.1021/acsnano.9b03631 31356751

[B65] Rahmana Z. Sung B. H. Yi J. Y. Bui le M. Lee J. H. Kim S. C. (2014). Enhanced production of n-alkanes in *Escherichia coli* by spatial organization of biosynthetic pathway enzymes. J. Biotechnol. 192 Pt A, 187–191. 10.1016/j.jbiotec.2014.10.014 25456061

[B66] Reifenrath M. Oreb M. Boles E. Tripp J. (2020). Artificial ER-derived vesicles as synthetic organelles for *in vivo* compartmentalization of biochemical pathways. ACS Synth. Biol. 9 (11), 2909–2916. 10.1021/acssynbio.0c00241 33074655

[B67] Sachdeva G. Garg A. Godding D. Way J. C. Silver P. A. (2014). *In vivo* co-localization of enzymes on RNA scaffolds increases metabolic production in a geometrically dependent manner. Nucleic Acids Res. 42 (14), 9493–9503. 10.1093/nar/gku617 25034694 PMC4132732

[B68] Sahtoe D. D. Praetorius F. Courbet A. Hsia Y. Wicky B. I. M. Edman N. I. (2022). Reconfigurable asymmetric protein assemblies through implicit negative design. Science 375 (6578), eabj7662. 10.1126/science.abj7662 35050655 PMC9881579

[B69] Sarin L. P. Hirvonen J. J. Laurinmäki P. Butcher S. J. Bamford D. H. Poranen M. M. (2012). Bacteriophage ϕ6 nucleocapsid surface protein 8 interacts with virus-specific membrane vesicles containing major envelope protein 9. J. Virol. 86 (9), 5376–5379. 10.1128/jvi.00172-12 22379079 PMC3347372

[B70] Sarma G. N. Kinderman F. S. Kim C. von Daake S. Chen L. Wang B. C. (2010). Structure of D-AKAP2:PKA RI complex: insights into AKAP specificity and selectivity. Structure 18 (2), 155–166. 10.1016/j.str.2009.12.012 20159461 PMC3090270

[B71] Sato K. Hamada M. (2023). Recent trends in RNA informatics: a review of machine learning and deep learning for RNA secondary structure prediction and RNA drug discovery. Brief. Bioinform 24 (4), bbad186. 10.1093/bib/bbad186 37232359 PMC10359090

[B72] Schlessinger J. (1994). SH2/SH3 signaling proteins. Curr. Opin. Genet. Dev. 4 (1), 25–30. 10.1016/0959-437x(94)90087-6 8193536

[B73] Shi Y. Wang D. Li R. Huang L. Dai Z. Zhang X. (2021). Engineering yeast subcellular compartments for increased production of the lipophilic natural products ginsenosides. Metab. Eng. 67, 104–111. 10.1016/j.ymben.2021.06.002 34153454

[B74] Sinclair J. F. Tzagoloff A. Levine D. Mindich L. (1975). Proteins of bacteriophage phi6. J. Virol. 16 (3), 685–695. 10.1128/jvi.16.3.685-695.1975 1159897 PMC354716

[B75] Siu K. H. Chen R. P. Sun Q. Chen L. Tsai S. L. Chen W. (2015). Synthetic scaffolds for pathway enhancement. Curr. Opin. Biotechnol. 36, 98–106. 10.1016/j.copbio.2015.08.009 26322735

[B76] Somasundaram S. Jeong J. Irisappan G. Kim T. W. Hong S. H. (2020). Enhanced production of malic acid by Co-localization of phosphoenolpyruvate carboxylase and malate dehydrogenase using synthetic protein scaffold in *Escherichia coli* . Biotechnol. Bioprocess Eng. 25 (1), 39–44. 10.1007/s12257-019-0269-1

[B77] Srinivasan P. Smolke C. D. (2020). Biosynthesis of medicinal tropane alkaloids in yeast. Nature 585 (7826), 614–619. 10.1038/s41586-020-2650-9 32879484 PMC7529995

[B78] Stitt B. L. Mindich L. (1983). Morphogenesis of bacteriophage phi 6: a presumptive viral membrane precursor. Virology 127 (2), 446–458. 10.1016/0042-6822(83)90157-5 6868372

[B79] Sun X. Yuan Y. Chen Q. Nie S. Guo J. Ou Z. (2022). Metabolic pathway assembly using docking domains from type I cis-AT polyketide synthases. Nat. Commun. 13 (1), 5541. 10.1038/s41467-022-33272-2 36130947 PMC9492657

[B80] Team Z. J. C. Chen M. (2015). RNA scaffold: designed to Co-localize enzymes. Methods Mol. Biol. 1316, 105–112. 10.1007/978-1-4939-2730-2_9 25967056

[B81] Tippmann S. Anfelt J. David F. Rand J. M. Siewers V. Uhlen M. (2017). Affibody scaffolds improve sesquiterpene production in *Saccharomyces cerevisiae* . ACS Synth. Biol. 6 (1), 19–28. 10.1021/acssynbio.6b00109 27560952

[B82] Tittes Y. U. Herbst D. A. Martin S. F. X. Munoz-Hernandez H. Jakob R. P. Maier T. (2022). The structure of a polyketide synthase bimodule core. Sci. Adv. 8 (38), eabo6918. 10.1126/sciadv.abo6918 36129979 PMC9491710

[B83] Tonikian R. Zhang Y. Sazinsky S. L. Currell B. Yeh J. H. Reva B. (2008). A specificity map for the PDZ domain family. PLoS Biol. 6 (9), e239. 10.1371/journal.pbio.0060239 18828675 PMC2553845

[B84] Tran K.-N. T. Kumaravel A. Hong S. H. (2023). Impact of the synthetic scaffold strategy on the metabolic pathway engineering. Biotechnol. Bioprocess Eng. 28 (3), 379–385. 10.1007/s12257-022-0350-z

[B85] Wang L. Sun Y. Lv D. Liu B. Guan Y. Yu D. (2020). Protein scaffold optimizes arrangement of constituent enzymes in indigoidine synthetic pathway to improve the pigment production. Appl. Microbiol. Biotechnol. 104 (24), 10493–10502. 10.1007/s00253-020-10990-5 33151367

[B86] Wang S. Z. Zhang Y. H. Ren H. Wang Y. L. Jiang W. Fang B. S. (2017a). Strategies and perspectives of assembling multi-enzyme systems. Crit. Rev. Biotechnol. 37 (8), 1024–1037. 10.1080/07388551.2017.1303803 28423958

[B87] Wang Y. Heermann R. Jung K. (2017b). CipA and CipB as scaffolds to organize proteins into crystalline inclusions. ACS Synth. Biol. 6 (5), 826–836. 10.1021/acssynbio.6b00323 28186716

[B88] Watson J. L. Juergens D. Bennett N. R. Trippe B. L. Yim J. Eisenach H. E. (2023). *De novo* design of protein structure and function with RFdiffusion. Nature 620 (7976), 1089–1100. 10.1038/s41586-023-06415-8 37433327 PMC10468394

[B89] Wei Q. He S. Qu J. Xia J. (2020). Synthetic multienzyme complexes assembled on virus-like particles for cascade biosynthesis in cellulo. Bioconjug Chem. 31 (10), 2413–2420. 10.1021/acs.bioconjchem.0c00476 33001630

[B90] Wei Q. Wang Y. Liu Z. Liu M. Cao S. Jiang H. (2022). Multienzyme assembly on caveolar membranes in cellulo. ACS Catal. 12 (14), 8372–8379. 10.1021/acscatal.2c01906

[B91] Weissman K. J. (2016). Genetic engineering of modular PKSs: from combinatorial biosynthesis to synthetic biology. Nat. Prod. Rep. 33 (2), 203–230. 10.1039/c5np00109a 26555805

[B92] Wong W. Scott J. D. (2004). AKAP signalling complexes: focal points in space and time. Nat. Rev. Mol. Cell Biol. 5 (12), 959–970. 10.1038/nrm1527 15573134

[B93] Wu J. Wang X. Xiao L. Wang F. Zhang Y. Li X. (2021). Synthetic protein scaffolds for improving R-(−)-Linalool production in *Escherichia coli* . J. Agric. Food Chem. 69 (20), 5663–5670. 10.1021/acs.jafc.1c01101 33983023

[B94] Xie S. S. Qiu X. Y. Zhu L. Y. Zhu C. S. Liu C. Y. Wu X. M. (2019). Assembly of TALE-based DNA scaffold for the enhancement of exogenous multi-enzymatic pathway. J. Biotechnol. 296, 69–74. 10.1016/j.jbiotec.2019.03.008 30885657

[B95] Xu Y. Wang X. Zhang C. Zhou X. Xu X. Han L. (2022). *De novo* biosynthesis of rubusoside and rebaudiosides in engineered yeasts. Nat. Commun. 13 (1), 3040. 10.1038/s41467-022-30826-2 35650215 PMC9160076

[B96] Yang D. Park S. Y. Lee S. Y. (2021). Production of rainbow colorants by metabolically engineered *Escherichia coli* . Adv. Sci. (Weinh) 8 (13), e2100743. 10.1002/advs.202100743 34032018 PMC8261500

[B97] Yang Z. Gao X. Xie H. Wang F. Ren Y. Wei D. (2017). Enhanced itaconic acid production by self-assembly of two biosynthetic enzymes in *Escherichia coli* . Biotechnol. Bioeng. 114 (2), 457–462. 10.1002/bit.26081 27543843

[B98] Yuan W. Jiang C. Wang Q. Fang Y. Wang J. Wang M. (2022). Biosynthesis of mushroom-derived type II ganoderic acids by engineered yeast. Nat. Commun. 13 (1), 7740. 10.1038/s41467-022-35500-1 36517496 PMC9748899

[B99] Zalatan J. G. Lee M. E. Almeida R. Gilbert L. A. Whitehead E. H. La Russa M. (2015). Engineering complex synthetic transcriptional programs with CRISPR RNA scaffolds. Cell 160 (1-2), 339–350. 10.1016/j.cell.2014.11.052 25533786 PMC4297522

[B100] Zhang J. Hansen L. G. Gudich O. Viehrig K. Lassen L. M. M. Schrubbers L. (2022). A microbial supply chain for production of the anti-cancer drug vinblastine. Nature 609 (7926), 341–347. 10.1038/s41586-022-05157-3 36045295 PMC9452304

[B101] Zhao C. Gao X. Liu X. Wang Y. Yang S. Wang F. (2016). Enhancing biosynthesis of a ginsenoside precursor by self-assembly of two key enzymes in Pichia pastoris. J. Agric. Food Chem. 64 (17), 3380–3385. 10.1021/acs.jafc.6b00650 27074597

[B102] Zhou Y. Dong J. Wang Q. (2023). Fabricating higher-order functional DNA origami structures to reveal biological processes at multiple scales. NPG Asia Mater. 15 (1), 25. 10.1038/s41427-023-00470-3

[B103] Zhou Y. J. Buijs N. A. Zhu Z. Gómez D. O. Boonsombuti A. Siewers V. (2016). Harnessing yeast peroxisomes for biosynthesis of fatty-acid-derived biofuels and chemicals with relieved side-pathway competition. J. Am. Chem. Soc. 138 (47), 15368–15377. 10.1021/jacs.6b07394 27753483

[B104] Zhu L. Y. Qiu X. Y. Zhu L. Y. Wu X. M. Zhang Y. Zhu Q. H. (2016). Spatial organization of heterologous metabolic system *in vivo* based on TALE. Sci. Rep. 6, 26065. 10.1038/srep26065 27184291 PMC4869064

